# Lipid-Related Domestication Accounts for the Extreme Cold Sensitivity of Semiwild and Tropic Xishuangbanna Cucumber (*Cucumis sativus* L. var. *xishuangbannanesis*)

**DOI:** 10.3390/ijms25010079

**Published:** 2023-12-20

**Authors:** Rui-Jing Zhang, Bin Liu, Shan-Shan Song, Radwa Salah, Chang-Jiang Song, Shi-Wei Xia, Qian Hao, Yan-Jun Liu, Yu Li, Yun-Song Lai

**Affiliations:** 1 College of Horticulture, Sichuan Agricultural University, Chengdu 611130, Chinasradwa461@gmail.com (R.S.);; 2 Hami-Melon Research Center, Xinjiang Academy of Agricultural Sciences, Urumqi 830091, China; liu.bin@xaas.ac.cn

**Keywords:** temperature stress tolerance, QTL mapping, structural variants, lipids, domestication

## Abstract

Xishuangbanna (XIS) cucumber (*Cucumis sativus* L. var. *xishuangbannanesis*) is a semiwild variety originating from low latitude tropic areas, and therefore shows extreme cold sensitivity and heat tolerance. Here, we mapped the quantitative trait loci (QTLs) that control the cold sensitivity and heat tolerance of XIS cucumber seedlings. Using bulked segregant analysis (BSA), we identified three QTLs (*HTT1.1*, *HTT3.1*, and *HTT3.2*, with a total length of 11.98 Mb) for heat tolerance and two QTLs (*LTT6.1* and *LTT6.2*, with a total length of 8.74 Mb) for cold sensitivity. The QTL *LTT6.1* was then narrowed down to a length of 641 kb by using kompetitive allele-specific PCR (KASP) markers. Based on structural variants (SVs) and single-nucleotide polymorphisms (SNPs), we found the *LTT6.1* is covered by a high divergent region including a 50 kb deletion in the XIS49 genome, which affects the gene structure of lipase *abhydrolase domain containing 6* (*ABHD6*, Csa_6G032560). Accordingly, there is a very big difference in lipid composition, but not in other osmoprotectants like free amino acids and fatty acids, between XIS49 and cultivated cucumber CL. Moreover, we calculated the composite likelihood ratio (CLR) and identified selective sweeps from 115 resequencing data, and found that lipid- and fatty-acid-related processes are major aspects in the domestication of the XIS group cucumber. *LTT6.1* is a particularly special region positioned nearby lipid-related selective sweeps. These studies above suggested that the lipid-related domestication of XIS cucumbers should account for their extreme cold sensitivity.

## 1. Introduction

Cucumber (*Cucumis sativus* L.) originated in the low-latitude Indian subcontinent 10.1 million years ago, and this species has since evolved environmental adaptations in accordance with a south-to-north dispersal. Taxonomically, *C. sativus* includes one cultivated variety and three wild/semiwild varieties (var. *hardwickii*, var. *sikkimensis*, and var. *xishuangbannanesis*) [1,2]. The results of a genomic structural analysis based on 115 resequencing data proposed that all cucumber germplasms can be divided into four groups based on geography: a Eurasian group (EU), an East Asian group (EA), a Xishuangbanna (XIS) group, and an Indian group (IN) [3]. All wild cucumber plants belong to the IN group, while the EU group and EA group contain only cultivated cucumber plants; the members of the XIS cucumber group are known as semiwild cucumber plants. Compared with other cucumber groups, the XIS cucumber group showed the narrowest geographic distribution, only around the borders of China and Southeast Asian countries [4]. XIS cucumber plants are characterized by known morphological characteristics such as their production of mango-like fruit with orange or yellow flesh, which is due to ß-carotene accumulation, and great efforts have been made to clarify the molecular basis of these distinct traits [5,6,7], short-day flowering habits, and sex expression instability [8,9].

Cultivated cucumber is a warm-season vegetable plant species whose optimum growth temperature is between 25 °C and 32 °C. XIS cucumber plants are always the most heat-tolerant and cold-sensitive in temperature stress evaluation of cucumber germplasms, and therefore display exhibit extreme heat tolerance and extreme cold sensitivity [10,11]. Nevertheless, studies of cucumber temperature stress tolerance usually use cultivated cucumbers as plant materials rather than XIS cucumbers. The first inheritance study of chilling resistance in 1992 suggested a single dominant gene [12], although some argued that it was the chloroplast genome that carried cold resistance genes [13]. Hence, many studies have involved quantitative trait locus (QTL) mapping, but no steady evidence supports candidate low temperature tolerance (LTT) genes [14,15,16,17,18,19,20]. Studies of reverse genetics have posited the positive regulation of G protein alpha-subunit (GPA) during the cold acclimation of cucumber via the CDL1–brassinolide (BR) pathway and cold-regulated COR413PM2–Ca^2+^–inducer of CBF expression (ICE)/C-repeat binding factors (CBF) pathway [21]. In contrast with cold tolerance, there are fewer studies about the heat response or heat resistance of cucumber plants [10,22,23].

The important role of osmoprotectants including polyols, sugars, amino acids, and betains in cold adaptation and cold tolerance have been well determined [24,25]. These low-molecular-weight compounds can stabilize the cellular structure and enzymes, scavenge reactive oxygen species and function as metabolic signals during abiotic stresses. Lipids play a critical role in the stabilization of the membrane system and it is now well known that the changes in lipid composition, especially unsaturated lipids, are directly correlated with the cold tolerance of plants [26]. A good example is FATTY ACIDS DESATURASE, which inserts unsaturated bonds into fatty acids and can enhance plant cold tolerance [27]. Today, lipidomics as detected using liquid chromatography mass spectrometry (LC–MS) are widely used to profile the role of lipid metabolism in cold tolerance [28].

In this study, we utilized QTL mapping, screened a possible domestication site, and assayed osmoprotectants including fatty acids, free amino acids and lipids during cold treatment. The combination of bioinformation tools, genetic analysis and physiological analysis provides new information about the molecular basis of the extreme cold sensitivity of XIS cucumbers.

## 2. Results

### 2.1. QTL Mapping of Seedling Heat Tolerance in XIS Cucumbers

**Figure 1:**
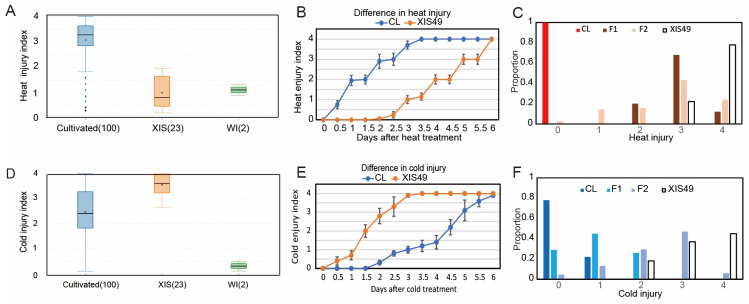
XIS cucumbers show extreme heat tolerance and cold sensitivity. (**A**) Comparison of heat tolerance in 23 XIS cucumbers, 100 cultivated cucumbers, and 2 wild cucumbers. (**B**) Gradual increases in heat injury were determined by their appearance during the heat treatment. (**C**) Heat tolerance of F1 and F2 population. (**D**) Comparison of cold tolerance in cultivated cucumbers, XIS cucumbers, and wild cucumbers. (**E**) Gradually increases in cold injury during cold treatment. (**F**) Cold tolerance of F1 and F2 population.

**Figure 2:**
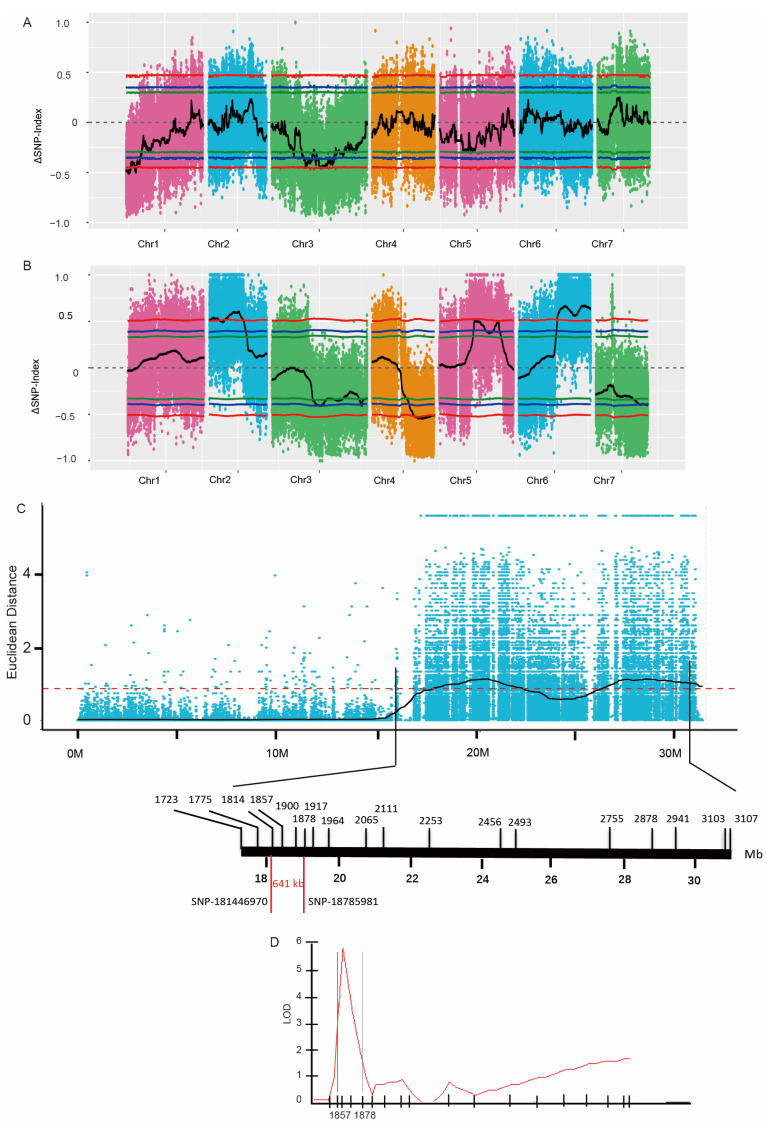
QTL mapping of HTT and LTT. (**A**) Distribution of ΔSNP index in bulked segregant analysis (BSA) of HTT. The red, blue and green lines indicate linkage threshold at 99%, 95% and 90% of confidence interval respectively. (**B**) Distribution of ΔSNP-index in BSA of LTT. The red, blue and green lines indicate linkage threshold at 99%, 95% and 90% of confidence interval respectively. (**C**) Fine mapping LTT6.1 based on SNP KASP markers. Red dashed line represents linkage threshold 0.84. (**D**) LOD profile of LTT6.1 detected on BC1 population (the recurrent parent, CL).

We compared the high-temperature tolerance (HTT) and LTT between XIS cucumbers (23 accessions) and other cucumbers (110 cultivated accessions and 2 Indian wild-type). The entire XIS variety showed extreme cold sensitivity and heat tolerance (Figure 1A,D). Interestingly, Indian wild cucumbers were strongly tolerant to both heat and cold stress. Bulked segregant analysis–DNA sequencing (BSA-seq) was performed to map the QTLs controlling HTT and LTT by using XIS49 and Chinese Long (CL, also known as 9930) (Table S5). The heat tolerance of XIS49 cucumbers is a recessive trait (Figure 1B,C). Reproducible QTL mapping results were obtained with the use of different markers or algorithms (Figure S2). There are two chromosome regions, the short arm of chromosome 1 and the central regions of chromosome 3, that were found to harbor significant (single-nucleotide polymorphism (SNP) index > 0.3) QTL signals according to the BSA data. The intersections of linked regions defined using different markers and algorithms were defined as the final QTLs. As a result, a total of three QTLs harboring 1092 genes with a total length of approximately 10 Mb were identified (Figure 2A and Table 1). At these QTLs, there were 1027 SNPs affecting the protein sequence of 478 genes (non-synonymous coding SNPs and open-reading-frame-altering SNPs), and 592 SVs were identified in the genic regions of 616 genes (Table S6). By applying a high-density kompetitive allele-specific PCR (KASP) marker, we tried to shorten the major HTT QTL, but the QTL signal was too weak in the fine mapping work.

### 2.2. QTL Mapping of Seedling Cold Sensitivity in XIS Cucumbers

During the cold treatment (6 °C), the XIS cucumber plants completely died on the third day, while the CL cucumber plants still survived on the sixth day (Figure 1E). Cold tolerance seems to be a dominant trait since the F1 population is resistant to cold stress (Figure 1F). Four chromosome regions were found to harbor significant (SNP index > 0.3) QTL signals (Figure 2B and S3). Two closely positioned QTLs (*LTT6.1* and *LTT6.2*) harboring 1340 genes with a total length of 8.7 Mb were identified (Table 1). At these QTLs, there were 908 SNPs affecting the protein sequence of 439 genes, and 462 SVs were identified in the genic regions of 360 genes (Table S7).

**Figure 3:**
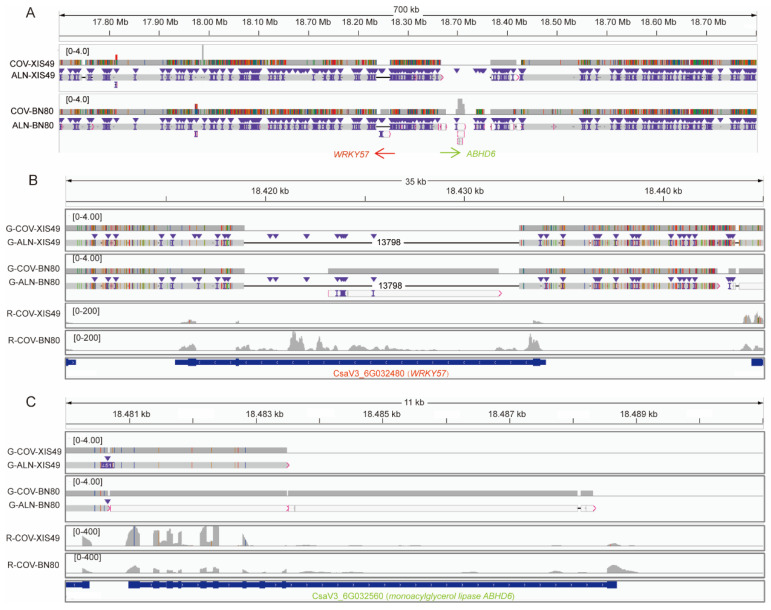
The QTL region LTT6.1 is rich in SNPs and SVs. Coverage track (COV) and alignment track (ALN) of XIS49 and BN80 genomes in the QTL region (**A**), WRKY57 gene (**B**), and ABHD6 gene (**C**). The inverted blue triangles indicate insertion markers. The color lines in coverage track and alignment track indicate mismatched bases.

To narrow the interval of major QTLs, novel SNP-based KASP markers were developed, and 300 BC1 seedlings were used for fine mapping. The QTL *LTT6.1* was narrowed to a region of 641 kb (between SNP18144697 and SNP18785981) (Figure 2C,D), a fragment that greatly differed between the parent genomes (Figure 3A). In this region, 10 SV genes presented differential transcript levels between XIS49 cucumbers and CL cucumber in the transcriptome (Table 2). The WRKY57 gene (CsaV3_6G032480.1) is totally suppressed in XIS49 cucumbers due to a large deletion in the intron (Figure 3B). Another candidate gene is abhydrolase domain containing 6 (ABHD6), which is a monoacylglycerol lipase (CsaV3_6G032560.1) (Figure 3C) with a distinct gene structure in XIS49 cucumbers due to an additional super-large deletion. Both WRKY57 and ABHD6 showed a response to cold treatment at the transcription level (Figure S4).

### 2.3. Lipid Metabolism Might Contribute to Differential Low-Temperature Tolerance

**Figure 4:**
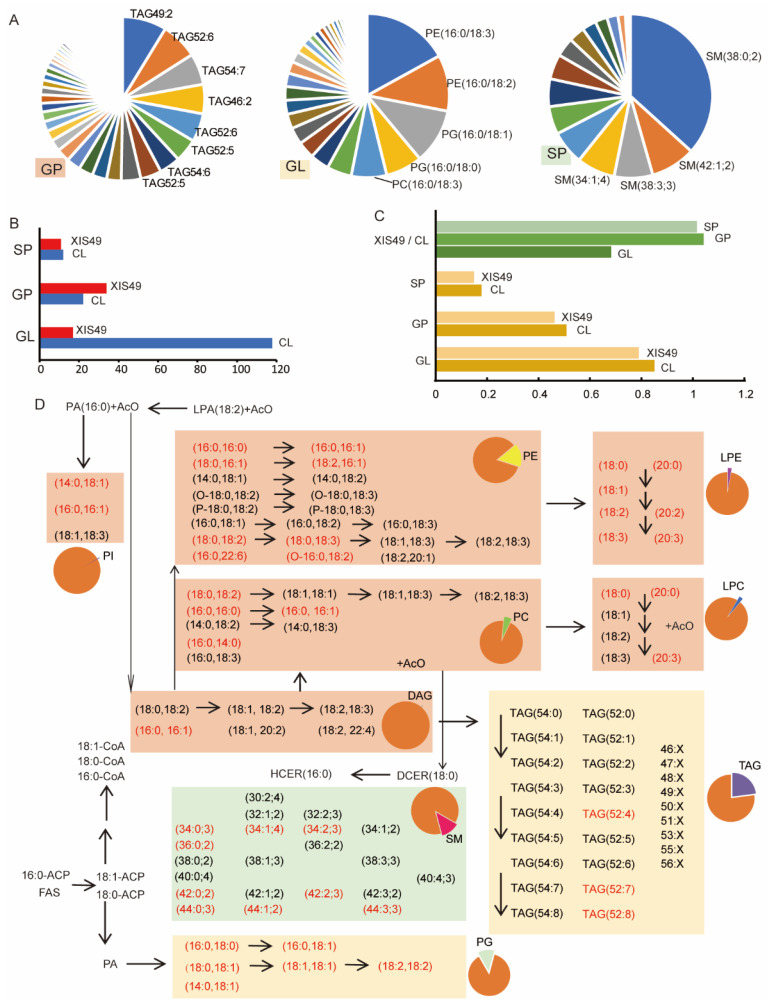
Lipid content and composition in XIS49 and CL cucumber after 24 h cold treatment. (**A**) Composition of detected glycero phosphatides (GPs), glycerolipids (GLs), and sphingolipids (SPs) in CL cucumber. (**B**) Difference in lipid accumulation after 24 h treatment. Red column, the number of lipids that showed higher contents in XIS49; blue column, the number of lipids that showed higher contents in CL. (**C**) The ratio of XIS49 to CL cucumber in total lipid content; proportion of unsaturated lipids. (**D**) Presentation of differentially accumulated lipid species. The pie charts indicate the proportion of each lipid species in the total lipids. The black arrows indicate the conversion between lipid molecules. Lipids being in red and black indicate their significantly higher and lower contents in XIS49, respectively. PI, phosphatidylinositol; PE, phosphatidylethanolamine; LPE, lysophosphatidyl ethanolamine; PC, phosphatidylcholine; LPC, lysophosphatidylcholine; DAG, diacylglycerol; TAG, triglyceride fatty acid; SM, sphingomyelin; PG, phosphatidylglycerol.

Lipids are critical components of the cell membrane, and there is a consensus on their roles in cold tolerance [29,30]. To elucidate the physiological mechanism of XIS49 cucumber cold sensitivity, we took CL cucumber as the control and assayed leaf lipids under LT treatment via liquid chromatography–tandem mass spectrometry (LC–MS/MS). The nomenclature of lipid molecules refers to a shorthand and standardized presentation system, which annotates the number of carbon atoms and double-bond numbers [31]. As many as 220 lipids were detected; these lipids belong to 14 subgroups and 3 groups, namely glyceryl phosphatides (GPs), glycerolipids (GLs) and sphingolipids (SP) (Table S8). The triglyceride (TAG) fatty subgroup, which includes 129 lipids and constitutes 29.8% of the total lipid amount, is the most abundant in the GP group; the most abundant TAGs are TAG49:2, TAG52:6, and TAG54:7 (Figure 4A). For the GL group, PE (16:0; 3), PE (16:0/18:2), and PG (16:0/18:1) were the most abundant; SM (38:0; 2) was predominant in the SP group (Figure 4A).

The leaves of the CL and XIS49 cucumber during the cold treatment presented totally different lipid compositions since all the lipids detected were significantly different (*p* < 0.5), and 142 showed highly significant differences (*p* < 0.01). Interestingly, the overwhelming majority of GL lipids, but not GPs and SPs, showed lower contents in the XIS49 cucumber than in the CL cucumber (Figure 4B). The total number of GLs in XIS49 cucumber was only 70% of that in CL cucumber; the ratio of unsaturated lipids was much higher in the CL cucumber than in the XIS49 cucumber (Figure 4C). The content change in each lipid was illustrated in a metabolic pathway map (Figure 4D). The contents of PG and lysophosphatidylethanolamine (LPE) were higher in XIS49 cucumber than in CL cucumber, which was very different from those of other lipids. In contrast, the CL cucumber accumulated many more TAGs than the XIS49 cucumber. For the other lipid subgroups, there were no consensus changes within each subgroup. Nearly all the TAGs that accumulated at relatively low levels are unsaturated lipids, which may account for the extreme cold sensitivity of XIS cucumbers.

### 2.4. Role of Fatty Acid and Free Amino Acids in Cold Sensitivity of XIS49 Cucumber

**Figure 5:**
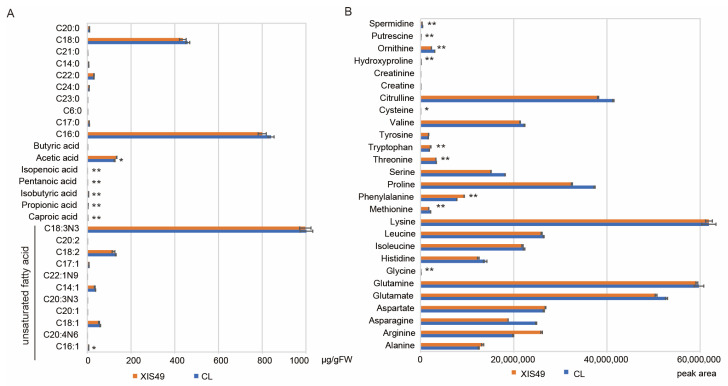
Content and composition of fatty acids and free amino acids in XIS49 and CL cucumber after 24 h cold treatment. (**A**) Fatty acids. (**B**) Free amino acids. * *p* < 0.05 and ** *p* < 0.01 indicate significant differences between XIS49 and CL.

The major role of lipids in cold resistance is as an osmoprotectant, while fatty acids (FAs) and free amino acids (FAAs) are two other osmoregulators. A total of 28 FAs were detected in the cucumber leaves via liquid chromatography–mass spectrometry (LS/MS), including 11 unsaturated FAs (Figure 5A). The nomenclature of fatty acids also refers to the standardized presentation system [31]. The C18:0, C16:0, and C18:3N3 FAs were the most abundant in the cucumber leaves and accounted for 90% of the detected FAs. There were seven FAs whose content significantly differed between XIS49 and CL cucumber. However, among these FAs, only acetic acid was somewhat abundant, and only one (palmitoleic acid) was an unsaturated FA. This result indicates the lesser importance of fatty acids in cucumber cold tolerance. A total of 27 FAAs were detected (Figure 5B). There were 22 FAAs whose content significantly differed between XIS49 and CL cucumber, among which the content of 16 FAAs was greater in CL cucumber than in XIS49 cucumber. Spermidine, asparagine, and ornithine are the top three FAAs that were more abundant in the CL cucumber than in the XIS49 cucumber, while putrescine, arginine, and phenylalanine are the top three whose contents were lowest in the CL cucumber. The proline content was 15% higher in CL cucumber than in XIS49 cucumber.

### 2.5. SVs Underlie the Domestication of XIS Cucumbers

To identify the role of SVs during cucumber domestication, we first analyzed the genomic selection region of XIS49 by calculating the composite likelihood ratio (CLR). There were 209 selective sweep (top 1%) regions distributed in all chromosomes except for chromosome 2 (Table S9). A total of 2529 SVs were identified in these regions of domestication-associated SVs (dSVs), which were associated with 1034 protein-coding genes (Table S10). The genetic function of some dSVs has been thoroughly clarified. For example, a variation in the promoter of GLABROUS 3 (CsaV3_6G049180) results in fewer trichomes in XIS49 cucumber [32].

**Figure 6:**
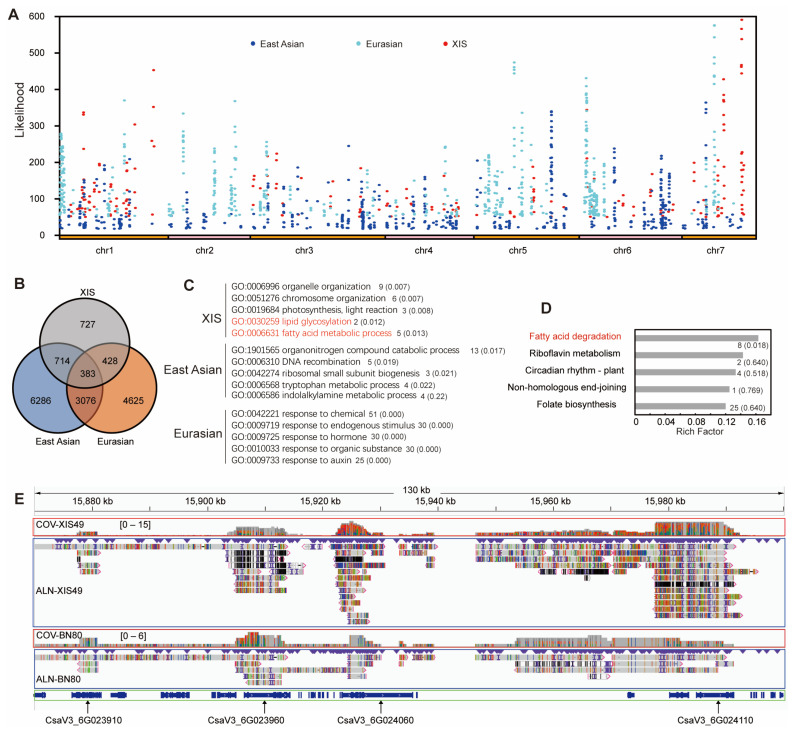
Lipid-related domestication in XIS cucumbers. (**A**) Selective sweep analysis of XIS group, EA group, and EU group by using resequencing reads of four groups of 115 cucumber accessions. IN group cucumbers were taken as a reference. Threshold value of domestication signal, top 1% composite likelihood ratio (CLR) value and 5% *p*-value. (**B**) Genome-specific selective sweeps were represented using Venn diagrams. (**C**) Top five GO terms in GO enrichment analysis by using XIS-, East-Asian-, and Eurasian-specific selective sweep genes. Gene number and *p*-value (in parentheses) were shown for each term. Lipid and fatty acid related GO terms were colored in red. (**D**) Top five pathways in KEEG enrichment analysis by using XIS-specific selective sweep genes. Gene number and Q-value (in parentheses) were shown for each pathway. Fatty acid related pathway was colored in red. (**E**) The four dSV genes (enoyl-CoA hydratase/3-hydroxyacyl-CoA dehydrogenase, EHHADH) enriched in fatty acid degradation pathway are clustered on chromosome 6 and show abundant diversity in gene sequence. COV, coverage track of targeted genome (XIS49 and BN80) to the reference genome CL. ALN, alignment track of targeted genome (XIS49 and BN80) to the reference genome CL. The inverted blue triangles indicate insertion markers. The color lines in coverage track and alignment track indicate mismatched bases. The mapped reads to the reference sequence were shown with their orientation below the alignment track. The color of the reads indicate reads quality, referring to published IGV for details [34].

Resequencing data of 115 cucumber accessions were released previously [3]. Then, selective sweep regions were identified via a genomic comparison of the XIS, EU, and EA cucumber groups with the wild IN group by using the resequencing data (Figure 6A). Interestingly, the dSV-genes are very different among XIS, EU and EA cucumbers, indicating that they underwent different domestication process (Figure 6B). Notably, we identified 24 dSV genes that participate in lipid-related processes, such as lipid metabolism, lipid binding, and lipid transformation, which may account for the large differences in performance between XIS49 cucumbers and cultivated cucumbers under cold treatment (Figure S5). There was an XIS-specific significant enrichment of dSV genes involved in fatty acid (FA) and lipid metabolism, which is distinct to the East Asian and Eurasian group (Figure 6C,D). For example, the lipase (CsaV3_7G034070) gene and alcohol dehydrogenase (CsaV3_6G031760) gene were not expressed in the XIS49 cucumbers, which is probably due to the highly diverged region (HDR) in their promoters (Figure 6E). These two genes are reported to play crucial roles in low-temperature tolerance [33].

## 3. Discussion

Cucumber plants are sensitive to both HT and LT. There are many reports on the exploration and utilization of temperature-stress-tolerant germplasms. Generally, approximately 5–10% of cucumber germplasms are highly tolerant to temperature stress [10,11,35]. Great efforts have been made to map the QTLs or genes controlling LTT [14,19,36,37] or HTT [10,38,39]. No consensus on the QTLs until now has been reported, which is probably due to the differences in plant materials and mapping strategies used. Moreover, none of those studies included XIS cucumbers despite this variety displaying extreme heat tolerance and cold sensitivity. Here, HTT QTLs were located on chromosome 3 and LTT QTLs at chromosome 6, which are close (not the same) to the QTLs reported previously [14]. Essentially, there is a major QTL on chromosome 6, but the detailed candidate gene is still unclear. Notably, *LTT6.1* is in fact positioned in a seriously polymorphic region. For *WRKY57*, the first intron is nearly entirely deleted in both XIS cucumbers (XIS49 and BN80), which results in expression suppression (Figure 3). *WRKY57* positively regulates drought tolerance and cold tolerance in Arabidopsis [40,41,42]. The *ABHD6* gene has 10 CDS in CL cucumber, but the 9th intron and the 10th CDS are lost in both XIS cucumbers. Although the expression level seems unaffected by the deletion, the protein function must be seriously affected. Monoacylglycerol lipase catalyzes the hydrolysis of monoglycerides into glycerol and fatty acids, whose expression is induced by cold and other abiotic stresses [43]. Although we proposed WRKY57 and ABHD6 to be the candidate genes, it is more likely that multiple genes and a complex genetic mechanism on chromosome 6 may control cold tolerance in cucumbers.

In many reported studies, temperature stress tolerance was derived from a special material with natural variation, mutation, or genetic modification. Here, extreme cold sensitivity and extreme heat tolerance are commonalities in XIS cucumbers, a special group originating from a narrow tropic area. XIS cucumbers are absolutely monophyletic and homogeneous, which is quite different from other cucumbers [3]. We found a large difference in the lipid metabolites between XIS49 and CL cucumber. XIS49 accumulated fewer glycerolipids but higher levels of PG and LPE. Glycerolipids are the major components of membranes; moreover, glycerolipids also function in the release of signaling molecules such as lysophospholipids in the temperature stress response [44]. TAGs and diacylglycerides (DAGs), the two glycerolipids identified in this study, accounted for the largest proportion of the total lipid contents in cucumber plants. Notably, most major glycerolipids are unsaturated, but XIS49 has a relatively low degree of unsaturated glycerolipids. Therefore, large differences in the TAG content and TAG saturation might account for the cold sensitivity of XIS49 cucumbers, although further investigation is required. PG is the only major phospholipid in thylakoid membranes and plays a specific role in photosynthetic electron transport [45]. A higher content of desaturated PG is suggested to be a very early indicator of the increased chilling sensitivity of plants [46,47]. Changes in TAGs, PG, and LPEs have been observed in many studies profiling the response to temperature changes, which may be the reason for or the result of temperature acclimation [48,49]. In addition to the changes in total contents, a delicate regulatory system that can switch between different lipid pathways was reported in Arabidopsis [50].

No fine mapping work of high quality until now has identified the genes controlling cold/heat tolerance in cucumbers. Temperature tolerance adjudgment of a single seedling is of low accuracy and therefore it is hard to find recombinant plants with high confidence. Further narrowing down the QTL regions of temperature stress tolerance relies on a more reliable judgment method without subjective influence, an accurate method to quantify the tolerance, and a fixed population to avoid error variation. Moreover, these reported QTLs of temperature tolerance usually have only minor effects, which can explain <20% of the trait variation in most cases. Since temperature adaptation is a main aspect for species survival, it must be the result of long-term evolution. Minor QTLs, cytoplasmic inheritance, and epigenetic mechanisms may also be involved in the cold tolerance regulation of cucumbers [18,51,52].

## 4. Materials and Methods

### 4.1. Plant Materials

The XIS49 were collected from Wenshan Prefecture, Yunnan Province, China, and were purified using self-crossing 6 times. Cucumber CL (also known as ‘9930’) is the sequencing material, with its updated genome assembly released first in 2009 and recently again in 2019. F2 and BC1 populations derived from a cross between XIS49 and CL were used for the BSA-seq and fine mapping, respectively.

### 4.2. High Temperature and Low Temperature Treatment

For the HT treatment, all the plants were initially grown in plastic pots under normal conditions—temperature at 25 °C, a photoperiod of 12 h/12 h, and a light intensity of 800 µmol/m^2^/s. The seedlings with four unfolded leaves were then moved into growth chambers for a two-day acclimation under 25 °C, followed by an HT treatment of 40 °C. The light conditions were the same (photoperiod of 12 h/12 h, and light intensity of 800 µmol/m^2^/s). The heat injury (HI) of each seedling was evaluated every 12 h. There were 5 grades of HI, and the HTT traits of each seedling was evaluated based on the HI value at 72 h (Figure S1).

For the LT treatment of seedlings, seedlings grown under a normal temperature at 25 °C in the culture room were then moved into a growth chamber for a short-term LT treatment (6 °C) when the fourth leaf began unfolding. The chilling injury (CI) of each seedling was evaluated every 12 h. There were 5 grades of (CI) values, and the LTT traits of each seedling was evaluated based on the CI value at 48 h (Figure S1). The light conditions were the same as described in the HT treatment.

### 4.3. Detection of Genomic Variants

Whole-genome sequence alignment of the newly assembled genome against the CL cucumber genome was performed by using the NUCmer program implemented in the software package MUMmer v4.0.0 with the options “-max -l 40 -g 90 -b 100 -c 200” [53]. The resulting alignments were filtered with a minimum alignment identity of 90 and a minimum alignment length of 100. Structural rearrangements and local variations were identified using SyRI v1.1 [54]. The gene copy numbers and gene families between assemblies were identified using OrthoFinder v2.3.14 [55].

### 4.4. QTL Mapping Using BSA-Seq

For the temperature stress tolerance, the F2 (CL × XIS49) population was used, and the population size for each QTL mapping work was 350. Each of the two extremely phenetic tools was constructed by equally mixing DNA from 30 seedlings. For QTL mapping of the hypocotyl length under low light, we used the same F2 population comprising 300 individuals. We pooled 50 individuals with extremely long hypocotyls and another 50 individuals with extremely short hypocotyls.

For the BSA-seq data processing, the DNA samples of the two parents and those of the two bulks in each QTL mapping system were subjected to whole-genome resequencing using the Illumina HiSeq platform. Duplicate clean reads were removed using the Picard tool (http://sourceforge.net/projects/picard/, accessed on 24 June 2021), and GATK v4.2.0.0) was used to perform local realignment and base recalibration to ensure the accuracy of the SNP detection [56]. The SNP loci between the test samples and reference genome were obtained using the GATK software according to the best practices listed on the GATK website (https://www.broadinstitute.org/gatk/guide/best-practices.php, accessed on 24 June 2021). All the SNPs between the test samples were summarized according to the alignment results of the test samples and the reference genome. The Euclidean distance (ED) [57] and Δ index [57,58,59] were subsequently calculated to identify the candidate regions of the genome associated with each target trait. The intersection of the linked regions defined using both algorithm methods (the ED and Δ index) and both marker types (SNPs and InDels) were defined as the final QTLs.

### 4.5. Fine Mapping Using SNP KASP Markers

To narrow the major QTLs of *LTT6.1*, *SH3.1*, and *SH6.1*, 23 kompetitive allele-specific PCR (KASP) markers were developed to determine the genotypes and a BC1 (with the recurrent parent, CL) population comprising 300 individuals was used (Table S1). Soft JoinMap4 was used to estimate the genetic distance, the logarithm of odds (LOD) values, and, ultimately, the *LTT6.1* QTL.

### 4.6. Differentially Expressed Genes (DEGs)

Differentially expressed genes (DEGs) between XIS49 and CL on the identified QTL *LTT6.1* were primarily determined based on RNA-seq reads (downloaded from NCBI under BioProject ID PRJNA817708). And quantitative real-time PCR (qRT-PCR) was carried out to further profile their response during the cold treatment. The leaf samples were collected at 0 h, 8 h, 16 h, 24 h, and 32 h in the cold treatment. The total RNA was extracted using the MolPure TRIeasy Plus Total RNA Kit (Yeasen Biotechnology, Shanghai, China). Reverse transcription was performed using Hifair V one-step RT-gDNA digestion SuperMix for qPCR (Yeasen Biotechnology, Shanghai, China). For the qRT-PCR, the PCR reagent was SYBR Prime qPCR Kit (Fast HS) (BioGround Biotechnology, Chongqing, China), and the apparatus was the Bio-Rad CFX96 (Bio-Rad, Hercules, CA, USA). The inner reference gene was ubiquitin (Gene ID: CsaV3_7G003730). All the primers are listed in Table S1.

### 4.7. Fatty Acid Detection Using GS–MS

Short-chain fatty acids (SCFAs) and long-chain fatty acids (LCFAs) were extracted and assayed using different methods. For the SCFAs, the standards were caproic acid (Sigma 99.5%), propionic acid (Sigma 99.0%), butyric acid (Sigma 99.0%), isobutyric acid (Sigma 99.0%), valeric acid (Sigma 98.0%), isovaleric acid (Sigma 99.0%), and caproic acid (Sigma 99.5%) (Table S2). For the LCFAs, a mix standard containing 37 fatty acid methyl esters (Nu-Check Prep, Elysian, MN, USA) was diluted to 1 μg/mL, 5 μg/mL, 10 μg/mL, 25 μg/mL, 50 μg/mL, 100 μg/mL, 250 μg/mL, 500 μg/mL, and 1000 μg/mL (Table S3). For the method of sample preparation, gas chromatography–mass spectrometry (GC/MS), and standard curve calculation, refer to the published paper [60].

### 4.8. Free Amino Acid Detection Using UPLC–MS/MS

The free amino acids (FAAs) were quantified using ultra-high performance liquid chromatography–tandem mass spectrometry (UPLC–MS/MS). The FAA detection referred to another method with some modifications [61]. The initial amount of leaf tissue for each sample homogenate was 80 mgFW. Ultra-performance liquid chromatography (UPLC) was applied by using the chromatograph LC-30A (Shimadzu, Kyoto, Japan), mass spectrometer QTRAP 5500 (AB SCIEX, Framingham, MA, USA), and ACQUITY UPLC BEH Amide column (particle size, 1.7 µm; 2.1 mm i.d.; 50 mm long; Waters, Milford, MA, USA). A total of 27 free amino acids were detected using this method (Table S4).

### 4.9. Targeted Lipidomics Detection Using UPLC–MS/MS

The total lipids were extracted following the method described by Bligh and Dyer with modifications [62]. The frozen leaf tissue (50 mgFW) was ground in liquid nitrogen and mixed with 1mL MTBE (methyl tert-butyl ether) solvent (MTBE–methanol = 3:1, *v*/*v*). The homogenate was shaken at 4 °C for 45 min, followed by ultrasonic extraction for 15 min in an ice bath. The homogenate was then mixed with 650 μL methanol–water (3:1, *v*/*v*), stabilized at room temperature for 10 min. The first layer after a 5 min centrifugation at 4 °C and 20,000× *g* was dried using a Termovap sample concentrator. The dry matter was resolved using isopropanol–methanol (1:1, *v*/*v*) for LC–MS analysis. A Nexera X2 LC-30AD (Shimadzu Corporation, Kyoto, Japan) HPLC system, QTRAP 5500 (AB SCIEX, Framingham, MA, USA) mass spectrometer, and ACQUITY UPLC BEH Amide column (particle size, 1.7 µm; 2.1 mm i.d.; 100 mm long; Waters, Milford, MA, USA). The detailed parameters of the HPLC system and mass spectrometry detection refer to a previously reported method [63].

### 4.10. Genome-Wide Selective Sweep

The selection signatures of the XIS cucumbers were identified using a genome-wide genetic diversity scanning method as previously described [64]. The resequencing data were downloaded from the NCBI Short Read Archive (SRA) under accession no. SRA056480 and the cross-population composite likelihood ratio test (XP-CLR) implemented in XP-CLR v. 1.0 was used [65,66]. Cucumbers of the XIS group and wild Indian group were the object population and reference population, respectively. Selective sweeps were determined by the CLR value, which is based on the site frequency spectrum (SFS) and allele frequency differentiation in the two populations. The whole genome was scanned in XP-CLR, choosing a sliding window of 1 Mbp at steps of 5 kbp. The XP-CLR settings were as follows: XPCLR -xpclr genofile1.txt genofile2.txt mapfile outputfile -w1 snpWin 0.01 gridSize 5000 chrN -p0 corrLevel 0.95; where genofile1.txt and genofile2.txt correspond to the object (XIS cucumbers) and reference (wild Indian cucumbers), respectively. The top 1% of the genome-wide XP-CLR values was used to delineate regions of interest. Neighboring windows were combined into one when the gap was less than 1 Mbp.

The selection signatures in the East Asian cucumbers, Eurasian cucumbers to wild Indian cucumbers were determined using the same method as with the XIS cucumbers. East Asian-, Eurasian-, and XIS-specific selective sweeps were subjected to further Gene Ontology (GO) and Kyoto Encyclopedia of Genes and Genomes (KEEG) enrichment analysis.

## Figures and Tables

**Table 1:** Quantitative trait loci (QTLs) controlling temperature stress tolerance and short hypocotyl under weak light.

	Chr.	Start	End	Size	Gene Num.	Strategy
*LTT6.1*	Chr6	17,700,000	21,750,000	4.05 Mb	476	BSA
*LTT6.1K*	Chr6	18,144,697	18,785,981	767.19 kb	123	KASP
*LTT6.2*	Chr6	26,380,000	31,070,000	4.69 Mb	864	BSA
*HTT1.1*	Chr1	34,193	5,059,683	5.03 Mb	703	BSA
*HTT3.1*	Chr3	14,529,644	15,494,828	0.97 Mb	89	BSA
*HTT3.2*	Chr3	20,308,431	26,288,906	5.98 Mb	319	BSA

LTT, low-temperature tolerance; HTT, high-temperature tolerance; BSA, bulked segregant analysis; KASP, kompetitive allele-specific PCR. LTT6.1K, the QTL defined using KASP markers.

**Table 2:** Differentially expressed SV genes at the locus *LTT6.1*.

Gene ID	Genome Position	SV Description	Gene Annotation
CsaV3_6G032330.1	18.31 Mb	Divergent intron	Proteasome subunit
CsaV3_6G032480.1	18.42 Mb	Divergent intron	WRKY57
CsaV3_6G032560.1	18.48 Mb	Divergent promoter and intron	Monoacylglycerol lipase ABHD6
CsaV3_6G032570.1	18.49 Mb	Divergent promoter and intron	Plasmodesmata callose-binding protein
CsaV3_6G033590.1	18.53 Mb	TE insertion in promoter	Unknown
CsaV3_6G033600.1	18.54 Mb	Divergent intron	Unknown

## Data Availability

Data are contained within the article or Supplementary Materials.

## Supplementary Materials

The following supporting information can be downloaded at https://www.mdpi.com/article/10.3390/ijms25010079/s1.
