# Supplementary material for: Lipid-Related Domestication Accounts for the Extreme Cold Sensitivity of Semiwild and Tropic Xishuangbanna Cucumber (Cucumis sativus L. var. xishuangbannanesis)
Source: Int J Mol Sci. 2023 Dec 20;25(1):79. doi: 10.3390/ijms25010079 (PMC10779220; doi:10.3390/ijms25010079)
Supplement: Supplementary file 1 [file ijms-25-00079-s001.zip › 4. Supplemental figures.pdf]

Supplemental figures

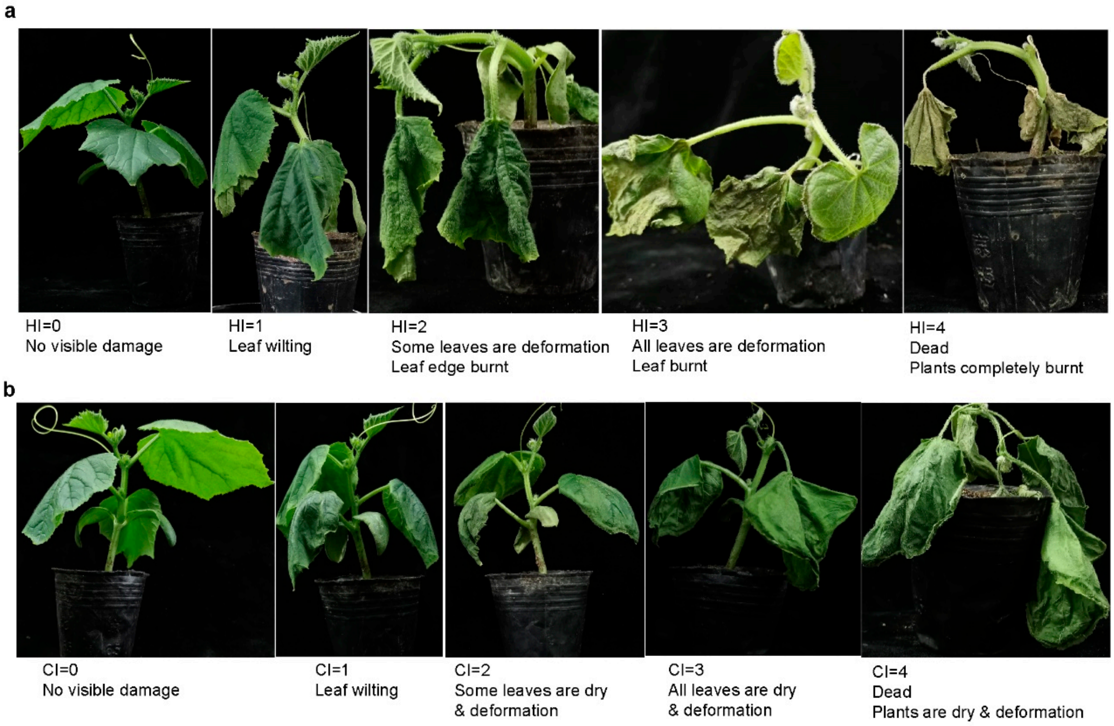

Figure S1. The standards to evaluate injury index in temperature-stress treatment. (**a**) High temperature treatment. (**b**) Low temperature treatment.

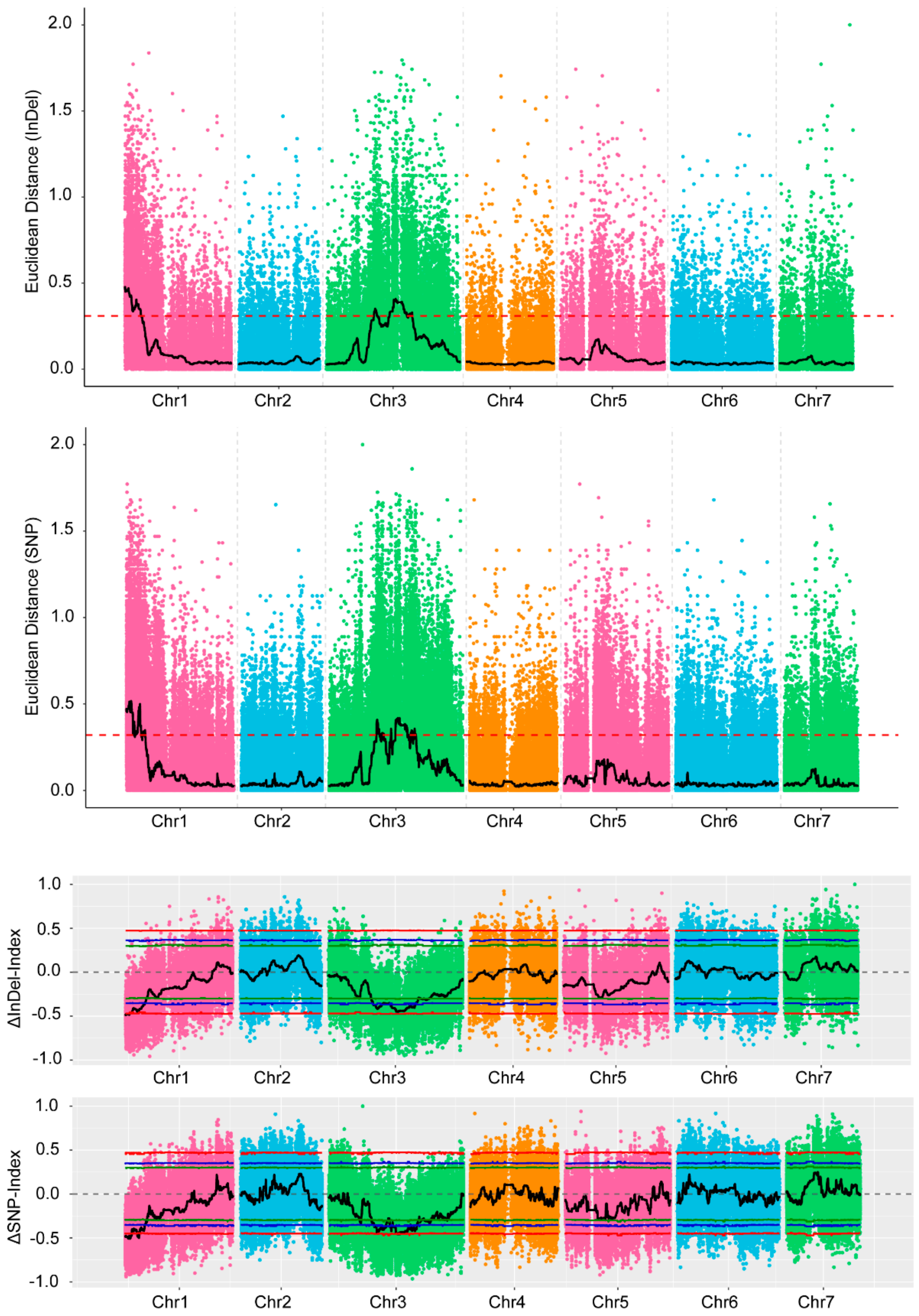

Figure S2. Distribution of Euclidean distance (ED), ΔSNP-index, or ΔInDel-index in bulked segregant analysis (BSA) of heat tolerance of seedling. A larger value of ED/ΔSNP-index/ΔInDel-index indicates a stronger linkage of the SNP site to target trait. In ED distribution, red dashed line represents linkage threshold 0.32. In ΔSNP-index and ΔInDel-index, the red, blue and green lines indicate linkage threshold at 99%, 95% and 90% of confidence interval respectively; Δindex=(cold-R bulk)-(cold-S bulk).

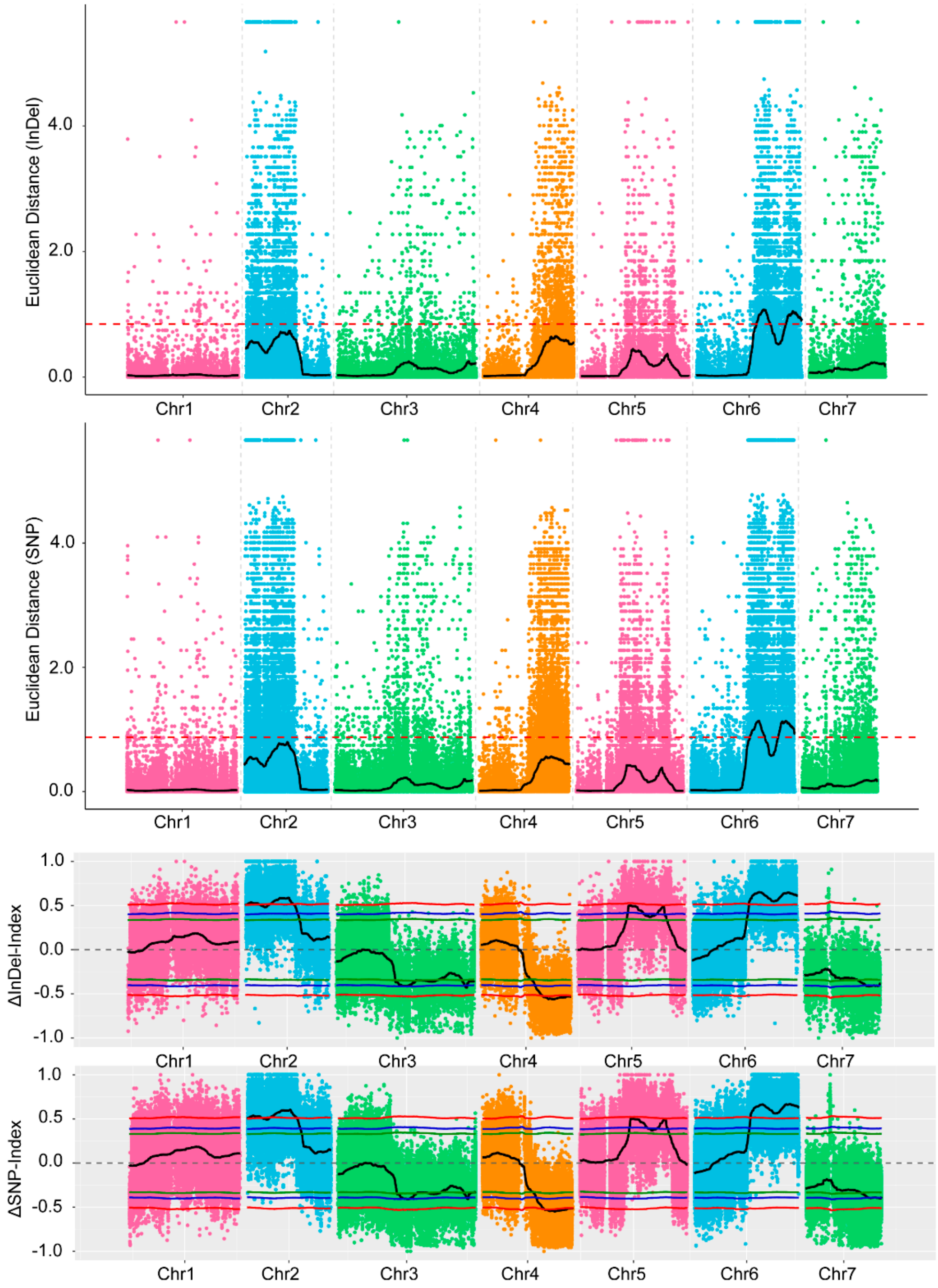

Figure S3. Distribution of Euclidean distance (ED), ΔSNP-index, or ΔInDel-index in bulked segregant analysis (BSA) of cold tolerance of seedling. A larger value of ED/ΔSNP-index/ΔInDel-index indicates a stronger linkage of the SNP site to target trait. In ED distribution, red dashed line represents linkage threshold 0.84. In ΔSNP-index and ΔInDel-index, the red, blue and green lines indicate linkage threshold at 99%, 95% and 90% of confidence interval respectively; Δindex=(cold-R bulk)-(cold-S bulk).

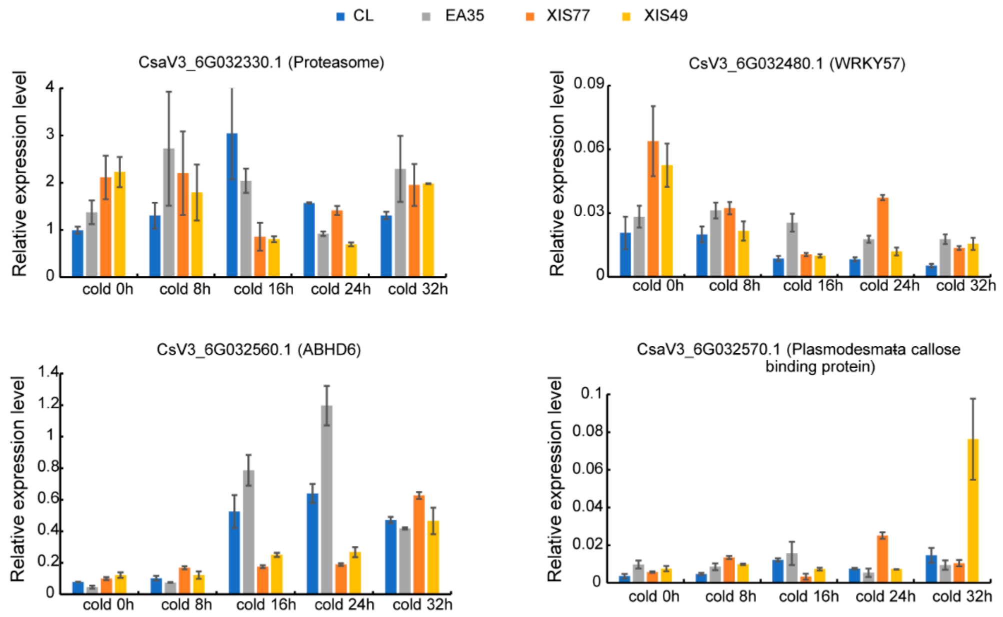

Figure S4. Expression profile of DEGs on QTL *LTT6.1* during the cold treatment. Expression level is quantified by qRT-PCR. Both CL and EA35 are East Asian cucumbers, and both XIS77 and XIS49 are XIS cucumbers.

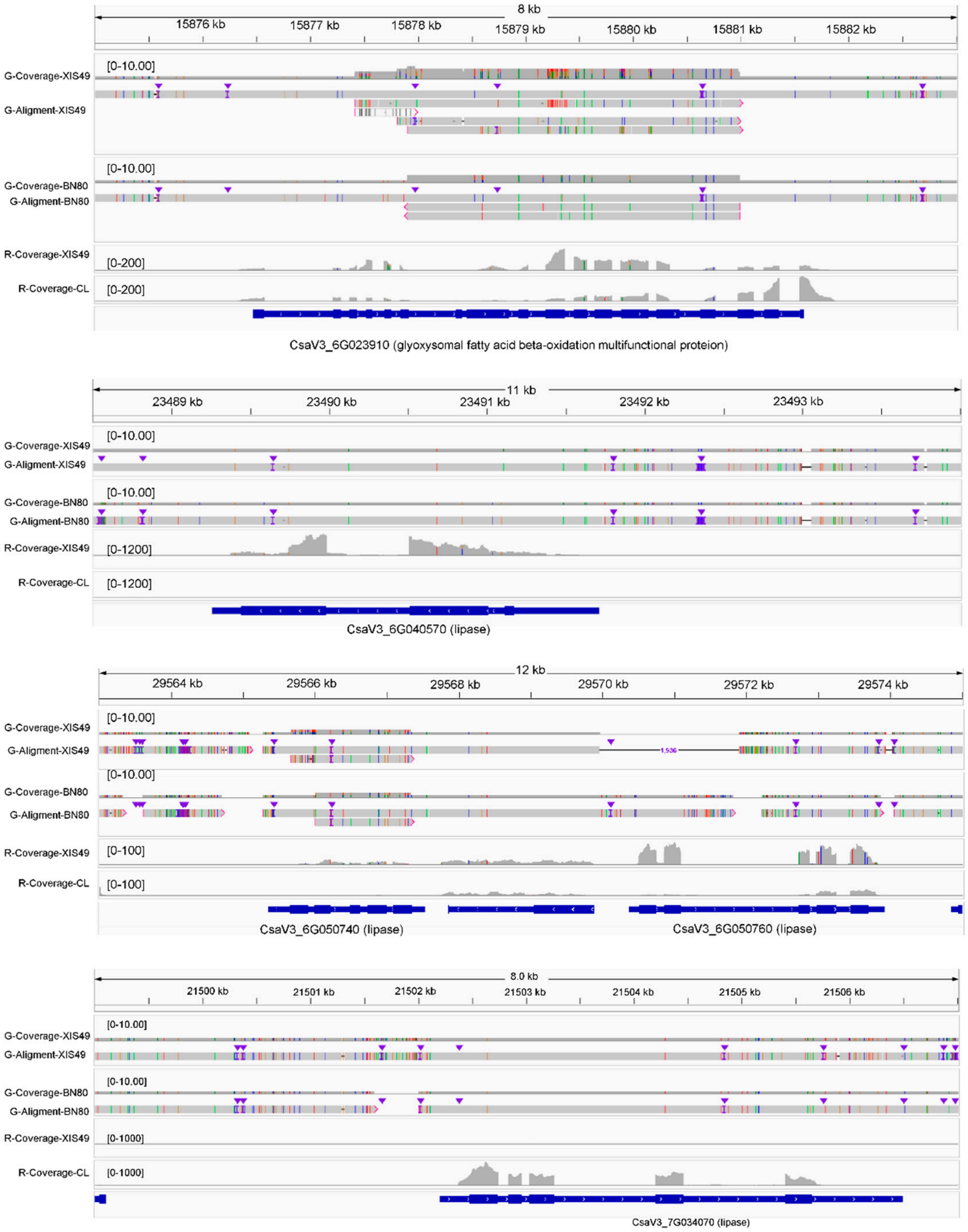

8 kb
15876 kb
15877 kb
15878 kb
15879 kb
15880 kb
15881 kb
15882 kb
G-Coverage-XIS49
G-Aligment-XIS49
G-Coverage-BN80
G-Aligment-BN80
R-Coverage-XIS49
R-Coverage-CL
[0-10.00]
[0-10.00]
[0-200]
[0-200]
CsaV3_6G023910 (glyoxysomal fatty acid beta-oxidation multifunctional proteion)
11 kb
23489 kb
23490 kb
23491 kb
23492 kb
23493 kb
G-Coverage-XIS49
G-Aligment-XIS49
G-Coverage-BN80
G-Aligment-BN80
R-Coverage-XIS49
R-Coverage-CL
[0-10.00]
[0-10.00]
[0-1200]
[0-1200]
CsaV3_6G040570 (lipase)
12 kb
29564 kb
29566 kb
29568 kb
29570 kb
29572 kb
29574 kb
G-Coverage-XIS49
G-Aligment-XIS49
G-Coverage-BN80
G-Aligment-BN80
R-Coverage-XIS49
R-Coverage-CL
[0-10.00]
[0-10.00]
[0-100]
[0-100]
CsaV3_6G050740 (lipase)
CsaV3_6G050760 (lipase)
8.0 kb
21500 kb
21501 kb
21502 kb
21503 kb
21504 kb
21505 kb
21506 kb
G-Coverage-XIS49
G-Aligment-XIS49
G-Coverage-BN80
G-Aligment-BN80
R-Coverage-XIS49
R-Coverage-CL
[0-10.00]
[0-10.00]
[0-1000]
[0-1000]
CsaV3_7G034070 (lipase)

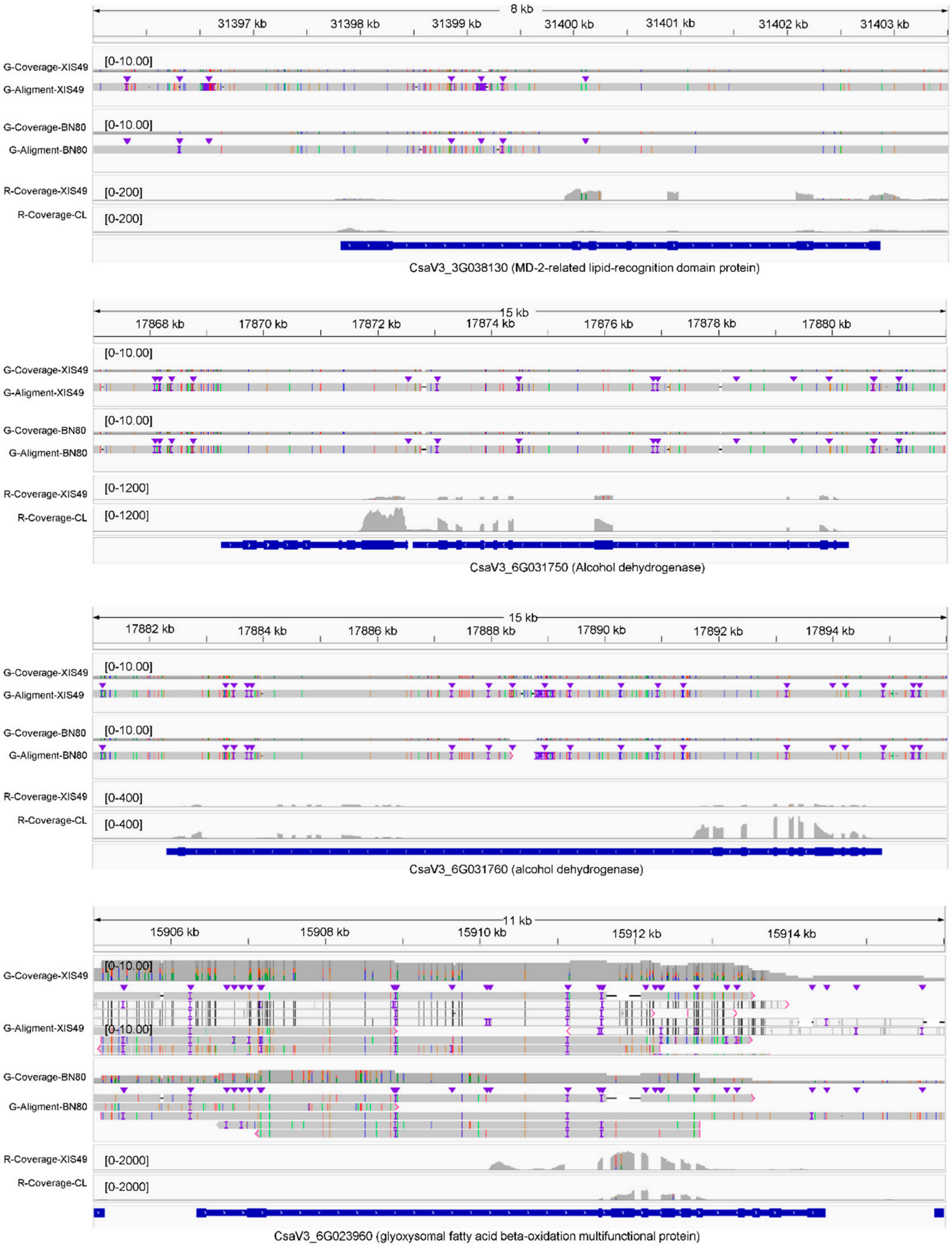

Figure S5. Domestication SV (dSV) regulate lipid metabolism genes. The difference in gene transcription is associated with SVs as demonstrated. SV and transcription level were vitalized by integrative genomics viewer (IGV). The inverted blue triangles indicate insertion markers. The color lines in coverage track and alignment track indicate mismatched bases.
